# Energy, exergy, and environmental performance of a solar dryer for orange slices across tray levels and thicknesses

**DOI:** 10.1038/s41598-025-23535-5

**Published:** 2026-01-31

**Authors:** Abdallah Elshawadfy Elwakeel, Awad Ali Tayoush Oraiath, Wajdi Aissa Mohammed Abdurraziq, András Székács, Omar Saeed, Mohamed Hamdy Eid, Mohammad S. AL-Harbi, Atef Fathy Ahmed, Aml Abubakr Tantawy

**Affiliations:** 1 Agricultural Engineering Department, Faculty of Agriculture and Natural Resources, Aswan University, Aswân, 81528 Egypt; 2 Department of Agricultural Engineering, Faculty of Agriculture, Omar Al Mukhtar University, P.O. Box 991, Al Bayda, Libya; 3 Agronomy Department, Faculty of Agriculture, Omar Al Mukhtar University, P.O. Box 991, Al Bayda, Libya; 4 Agro-Environmental Research Centre, Institute of Environmental Sciences, Hungarian University of Agriculture and Life Sciences, Páter Károly u. 1, Gödöllő, 2100 Hungary; 5 Doctoral School of Environmental Science, Hungarian University of Agriculture and Life Sciences (MATE), Páter Károly u. 1, Gödöllő, 2100 Hungary; 6 Institute of Environmental Management, Faculty of Earth Science, University of Miskolc, Miskolc-Egyetemváros, 3515 Hungary; 7 Geology Department, Faculty of Science, Beni-Suef University, Beni-Suef, 65211 Egypt; 8 Department of Biology, College of Science, Taif University, P.O. Box 11099, Taif, 21944 Saudi Arabia; 9 Food Science Department, Faculty of Agriculture, Beni-Suef University, Beni-Suef, 65211 Egypt

**Keywords:** Sustainable agriculture systems, Drying technology, Solar energy, Renewable energy, Citrus drying, Thermodynamic analysis, Engineering, Environmental sciences, Materials science

## Abstract

This research introduces the development of an automated forced and natural solar dryer (AFNSD) equipped with a photovoltaic-powered IoT technology, temperature-responsive control system that seamlessly alternates between natural and forced convection to improve efficiency and minimize energy consumption. In contrast to traditional fixed systems, it avoids both over-drying and product spoilage. The affordable, solar-driven design makes it ideal for off-grid communities. By combining drying kinetics analysis with economic and environmental evaluations, the system aligns with and promotes sustainability objectives. The thermodynamic performance and sustainability indicators were also evaluated. The developed AFNSD was used for drying orange slices at different tray positions (lower, middle, and upper), and three slice thicknesses (4, 6, and 8 mm). the obtained results showed that thinner orange slices (4 mm) placed on the lower trays reached the equilibrium moisture content more quickly, with an average drying time of about 13 h. In contrast, thicker slices (8 mm) positioned on the upper trays required the longest drying time, averaging around 25 h to reach the equilibrium moisture content. The thermodynamic analysis showed that the maximum energy efficiency of the solar collector (SC) ($\:{\eta\:}_{en,\:\:SC})\:$was about 70.98%. And the maximum exergy efficiency of the SC ($\:{\eta\:}_{ex,\:\:SC})\:$and the drying chamber (DCh) ($\:{\eta\:}_{ex,\:\:DCh}$) were about 21.93% and 43.64%, respectively. additionally, the sustainable indicators of both SC and DCh of the developed AFNSD, showed that the improved potential (IP) was in the range of 2.03 to 12.61 W in the SC and from 0.03 to 1.85 W in the DCh. The average waste energy ratio (WER) was 0.9 for the SC and 0.7 for the DCh. And the sustainability index (SI) ranged from 1.02 to 1.28 in the SC and from 1.2 to 1.77 in the DCh.

## Introduction

Oranges are among the most widely consumed fruits globally, appreciated for their flavor, high vitamin C content, dietary fiber, and beneficial phytochemicals like flavonoids and carotenoids^1–3^. These compounds provide antioxidant, anti-inflammatory, and heart-protective effects, and regular consumption of oranges has been linked to reduced risks of chronic diseases^4–6^. Beyond nutrition, oranges have medicinal properties, including anti-diabetic, anti-cancer, and antimicrobial effects^5,7^. Egypt is a leading orange producer and the world’s largest exporter, with average annual production of 2.3 million tons and exports valued at $661 million in 2019/2020. The Washington navel orange is the primary variety, grown on over 247,000 acres and valued for its taste and seedless nature^8,9^. However, oranges are highly perishable and susceptible to nutrient loss during storage^10,11^. Solar drying has emerged as an effective preservation method, offering a sustainable and energy-efficient alternative to traditional techniques^12–18^. These dryers use solar energy to speed up drying while retaining nutrients and sensory quality, and they offer better protection against microbial contamination and oxidation. In addition, solar dryers reduce dependence on fossil fuels and align with environmentally friendly agricultural practices, making them ideal for preserving oranges and similar fruits in sun-rich regions^19–21^.

The adoption of solar dryers is increasingly vital for promoting sustainability and clean energy in today’s world^22–25^. Solar dryers harness renewable solar energy to efficiently remove moisture from agricultural and food products, reducing reliance on fossil fuels and minimizing environmental impact^26–28^. This technology offers a clean, green alternative to traditional drying methods, which are often energy-intensive and contribute to greenhouse gas emissions^29–31^. Solar drying systems not only lower operational costs and carbon emissions but also improve product quality and reduce postharvest losses, supporting food security and sustainable agriculture^32–34^. As global demand for sustainable food processing grows, solar dryers represent a practical and scalable solution for clean energy utilization, especially in regions with abundant solar resources. Their widespread adoption can play a significant role in advancing climate-resilient energy practices and supporting a sustainable future^29–31,35^.

Solar dryers are categorized based on their method of utilizing solar energy, airflow mechanism, and structural configuration. The primary types include direct, indirect, mixed-mode, and hybrid solar dryers. In direct solar dryers, products are exposed directly to sunlight within an enclosed, transparent DCh. While simple and cost-effective, direct exposure may degrade product quality, especially for sensitive items^36–38^. Indirect solar dryers use a separate solar collector to heat air, which is then directed into a DCh. This design prevents direct sunlight from reaching the product, preserving color, flavor, and nutritional content—making it ideal for delicate foods^36–39^. Mixed-mode dryers combine both approaches, exposing the product to solar radiation while also using pre-heated air, thereby enhancing drying efficiency and uniformity^26,36,37^. Hybrid solar dryers incorporate additional heat sources, such as electric or biomass heaters, to maintain drying under low-sunlight conditions. Some hybrid systems also feature thermal energy storage for nighttime or cloudy operation, ensuring continuous and efficient drying^36,37,40^. Furthermore, In addition to energy source classification, solar dryers are also divided based on airflow mechanism into passive (natural convection) and active (forced convection) types. Passive dryers rely on natural air movement driven by temperature and pressure differences, making them energy-efficient and low-cost. However, airflow rates are less controllable, which may lead to longer drying times and inconsistent results. In contrast, active dryers use mechanical fans or blowers to force air through the system, ensuring uniform airflow, faster drying, and better control over temperature and humidity conditions. Though more complex and energy-dependent, active systems are generally preferred for high-value or large-scale drying operations^29,36^.

Photovoltaic–thermal (PVT) solar dryers combine solar thermal collectors with photovoltaic panels, enabling the simultaneous generation of heat and electricity for efficient and sustainable food drying. Recent research has focused on improving their performance through innovative designs and the integration of energy storage solutions. For example, a newly developed PVT dryer incorporating sand-filled thermal energy storage (TES) was evaluated for drying Moringa leaves under different airflow conditions, achieving notable efficiency gains^41^. Similarly, a hybrid PVT system with an evacuated tube collector, designed for cassava drying, demonstrated faster drying rates, greater energy savings, and better product quality compared to open sun drying^42^. Other advancements include mixed-mode and greenhouse-integrated PVT dryers, which have been assessed using MATLAB-based modeling and real-time experimental validation. Optimization techniques such as artificial neural networks (ANN) and computational fluid dynamics (CFD) have been applied to refine airflow patterns and predict drying behavior^43–46^. Some systems further extend functionality by incorporating heat pumps or thermoelectric generators (TEGs) to enhance energy recovery and storage capacity^47,48^. From an environmental and economic standpoint, PVT dryers substantially lower energy consumption, CO_2_ emissions, and operational costs compared to conventional drying methods. Economic evaluations have reported favorable payback periods ranging from 2.98 to 3.51 years, making these systems particularly suitable for small-scale and rural applications^49^.

Several recent studies have investigated the performance of solar dryers operating under natural, forced, and mixed convection systems, with a particular focus on energy and exergy analysis. These studies provide valuable insights into the thermodynamic behavior, efficiency, and sustainability of various dryer configurations. A summary of key research contributions in this area is presented below. Ekka and Muthukumar conducted an experimental study on the exergy efficiency and sustainability indicators of a forced convection mixed-mode solar dryer system used for drying cluster figs. The system incorporated two double-pass solar air collectors to enhance thermal performance. The investigation was carried out under varying conditions of air mass flow rates (ranging from 0.018 to 0.062 kg/s) and solar radiation intensities (120–750 W/m²). Results showed a significant increase in the exergy efficiency of the DCh, rising from 18.8% to 41.4% with increased air mass flow rate. The SI improved moderately, ranging from 1.26 to 1.71, indicating enhanced system performance. Conversely, the IP decreased with higher airflow, suggesting reduced room for further optimization under those conditions. Overall, the study demonstrated that optimizing air flow enhances energy utilization and system sustainability in mixed-mode solar drying^50^. Mugi et al. conducted a comparative energy and exergy analysis of natural indirect solar dryers (NISD) and forced convection solar dryers (FISD) while drying muskmelon slices. The study aimed to evaluate and compare system performance using key thermodynamic and sustainability metrics, including energy and exergy flows, SI, WAS, and IP. Results showed that the FISD outperformed the natural convection setup across all parameters. Collector energy efficiency improved from 58.5% in NISD to 66.37% in FISD, while drying efficiency rose from 9.39% to 12.11%. Exergy efficiency also increased notably—from 45.87% in NISD to 55.73% in FISD. Additionally, the SI saw a significant 60.69% increase with FISD, highlighting better environmental performance. WAS decreased by 18.52% in FISD, indicating reduced energy losses. The IP also dropped in the FISD system (from 0.11 to 29.1 W in NISD to 0.012–11.35 W), reflecting enhanced overall efficiency. These findings underscore the advantages of incorporating forced convection in indirect solar drying systems for improved energy utilization, reduced environmental impact, and higher product drying performance^51^. Chandramohan and Mugi carried out a comprehensive energy, exergy, economic, and environmental analysis comparing NISD and FISD modes of indirect solar dryers during the drying of guava slices. Their study evaluated system performance in terms of thermal and exergy efficiency, economic feasibility, and environmental impact, particularly focusing on CO_2_ mitigation. Results revealed that the FISD demonstrated superior overall performance. The solar collector efficiency increased from 56.05% in NISD to 65.37% in FISD, while drying efficiency improved from 5.42% to 6.84%. Exergy analysis showed that although the collector exergy efficiency was slightly higher in NISD (3.74%) compared to FISD (2.39%), the DCh exergy efficiency was notably better in FISD (57.03%) than in NISD (50.92%). From an economic standpoint, the payback period was significantly shorter for FISD at 1.38 years compared to 2.24 years for NISD, indicating faster return on investment. These findings highlight the advantages of forced convection in improving thermal and exergy performance, while also enhancing economic viability and reducing environmental footprint, making FISD a more effective and sustainable choice for drying high-moisture agricultural products like guava^52^. Payganeh et al. conducted an energy and exergy analysis of an indirect solar dryer using a dynamic mathematical model, which was validated through experimental data under varying air mass flow rates. Although the specific dried product was not mentioned, the study focused on optimizing key design and operational parameters such as air velocity, glass cover thickness, and solar collector length. The results revealed that the maximum exergy efficiency of the system reached 22%, highlighting moderate conversion of available energy into useful work. Increasing the air velocity significantly enhanced the exergy stream through improved heat transfer and system responsiveness, while simultaneously reducing exergy destruction and overall irreversibility within the system. The study demonstrated the effectiveness of dynamic modeling in capturing transient behavior and guiding the design of more efficient solar dryers. By fine-tuning operational variables, especially airflow conditions, the system’s energy utilization and thermal performance can be substantially improved, making it a valuable tool for optimizing indirect solar drying technologies across various applications^53^.

Traditional solar dryers function manually, depending on either natural convection or forced air circulation. Farmers are required to frequently supervise the process—adjusting vents, shielding produce from rain and pests, and managing drying conditions. Although these systems are affordable and simple to build, they often face challenges such as uneven drying, overheating, and residual moisture, which can de-grade product quality. In contrast, solar dryers combine both natural and forced convection, resulting in better thermal efficiency and more uniform drying. To address the limitations of traditional systems, the present study focused on developing an AFNSD equipped with sensors, microcontrollers, and electronic control circuits. This setup allows for automated regulation of the air suction fan mode (natural convection or forced air circulation) based on temperature, humidity, and airflow, reducing the need for manual intervention, enhancing energy efficiency, and maintaining consistent drying conditions. The automation significantly reduces labor demands and improves both drying performance and product quality. Additionally, the study aimed to evaluate the thermodynamic performance of the developed AFNSD through detailed energy and exergy analyses, along with the assessment of key sustainability indicators.

## Materials and methods

### Description of the developed AFNSD

**Fig. 1:**
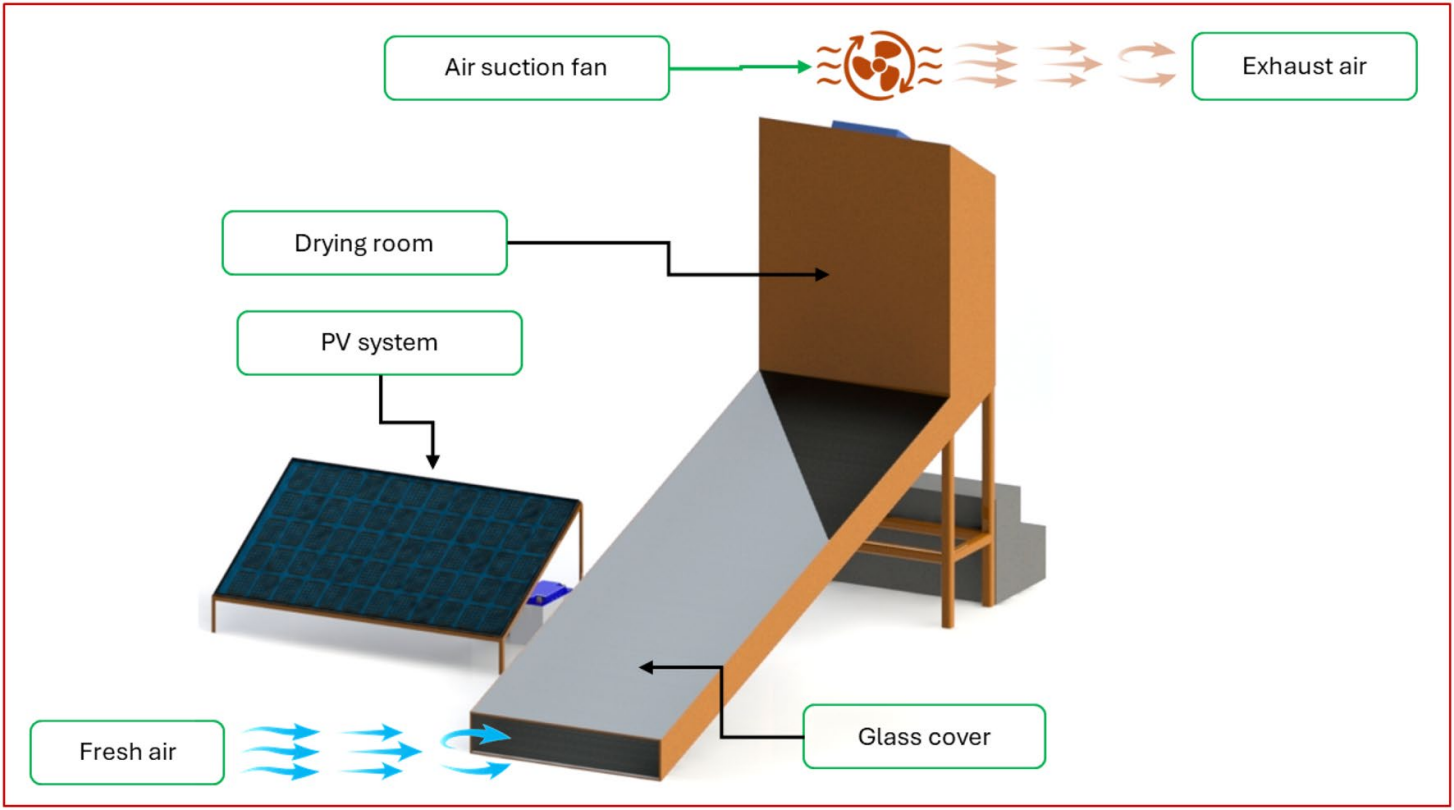
The main components of the developed AFNSD integrated with PV system.

Figure 1 illustrates the integrated layout and main components of the AFNSD, where it is developed to efficiently dry orange slices while conserving energy and maintaining product quality. The AFNSD comprises a flat plate solar collector, a DCh, a PV system, and an automated monitoring and control unit. The SC, measuring 300 cm × 100 cm × 20 cm, features a 3 mm-thick glass cover and a black corrugated aluminum absorber plate, insulated with a 3 cm layer of thermal wool for enhanced heat retention. The DCh includes eight trays (three used during this study as seen in Fig. 1), each 100 cm × 50 cm, equipped with four 12 V DC brushless fans to evenly circulate air and ensure uniform moisture content across the trays. The system integrates multiple sensors and control mechanisms for real-time environmental monitoring and intelligent system regulation. Five DHT22 (AM2302, accuracy: ±0.5 °C for temperature and ± 2–5% RH for humidity) sensors measure temperature and relative humidity at key points (inlet, outlet, and above each drying tray). A GL5506 LDR light sensor tracks solar intensity with resistance range: ~2–5 kΩ in light, while an infrared sensor monitors exhaust fan speed (LM393, Waveshare, China; high precision digital output ± 2%).

The core of the control system is the Arduino Mega 2560 R3 microcontroller, which processes sensor data and triggers system responses based on predefined thresholds. If temperature, humidity, or light intensity exceed critical values, the system activates forced convection through a DC fan connected to a 2-channel relay (SRD-05VDC-SL-C). Otherwise, it operates in passive mode using natural air movement. Additionally, a SIM900A GSM module sends SMS notifications in real time for system status or alerts, while a 16 × 2 LCD provides on-site display of environmental conditions.

**Fig. 2:**
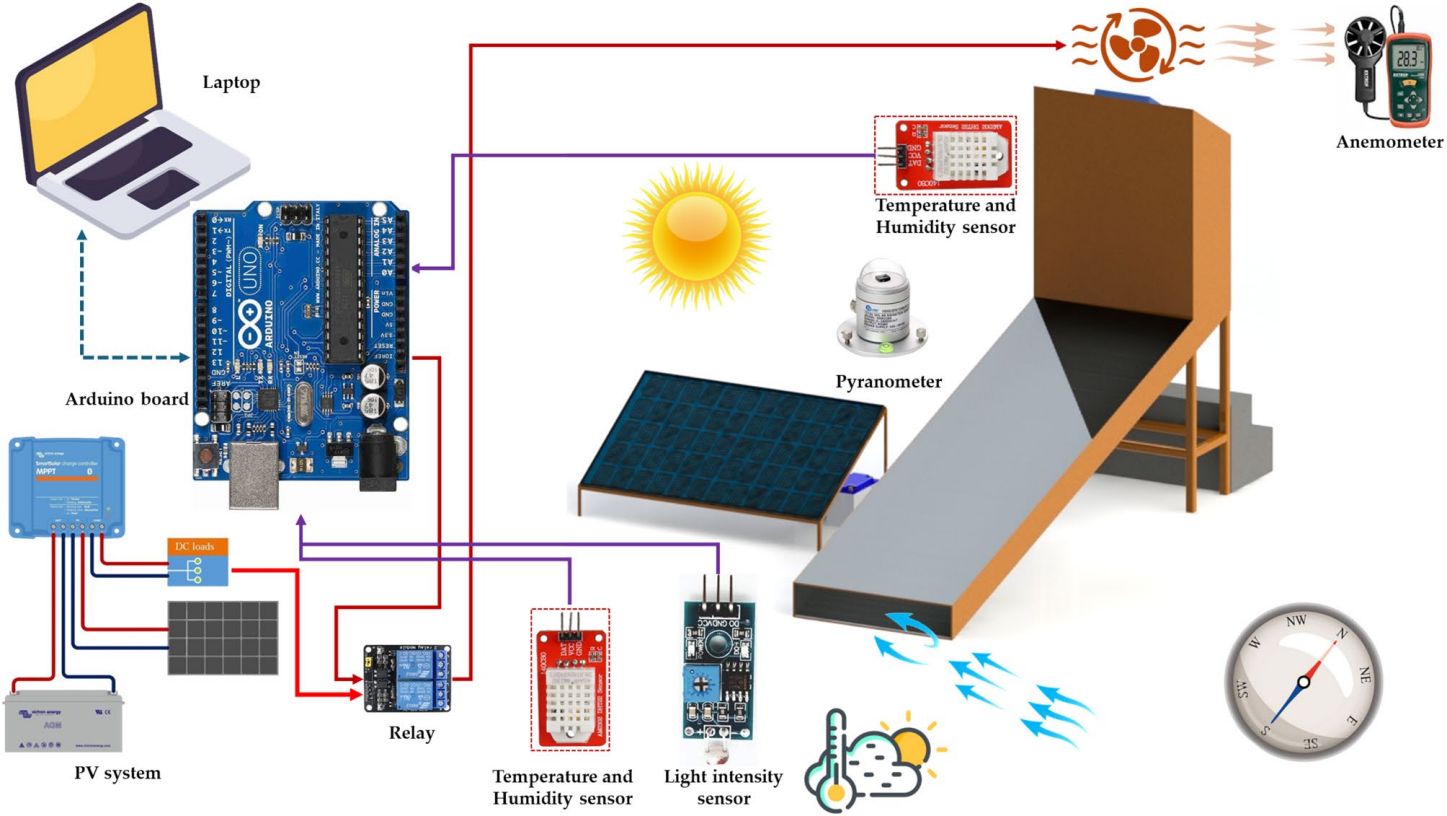
Rational operating and control map of the developed AFNSD.

The entire setup is powered by a 320 W polycrystalline solar panel (CS6X-320P), with energy managed by a 30 A charge controller (RBL-30 A) and stored in a 12 V, 70Ah battery, enabling off-grid functionality. The control algorithm, designed for energy efficiency, initiates system checks every five minutes, ensuring stable and responsive operation. This self-regulating solar drying solution offers a low-cost, adaptable design suitable for remote and energy-constrained environments. Figure 2 shows the rational operating and control map of the developed AFNSD.

The electronic components used in this study are certified by reputable manufacturers and have undergone rigorous calibration and quality assurance procedures at the production stage. To further validate their performance under local conditions, an additional calibration process was carried out prior to the experiment. This was conducted in collaboration with the Meteorology Unit at the Faculty of Agriculture and Natural Resources, Aswan University—the same location where the experimental work was performed. The purpose of this step was solely to verify the accuracy, stability, and reliability of the instruments before commencing measurements. Once field calibration confirmed proper functionality, the components were operated in accordance with the developed control system, without further on-site recalibration during the experimental phase.

**Fig. 3:**
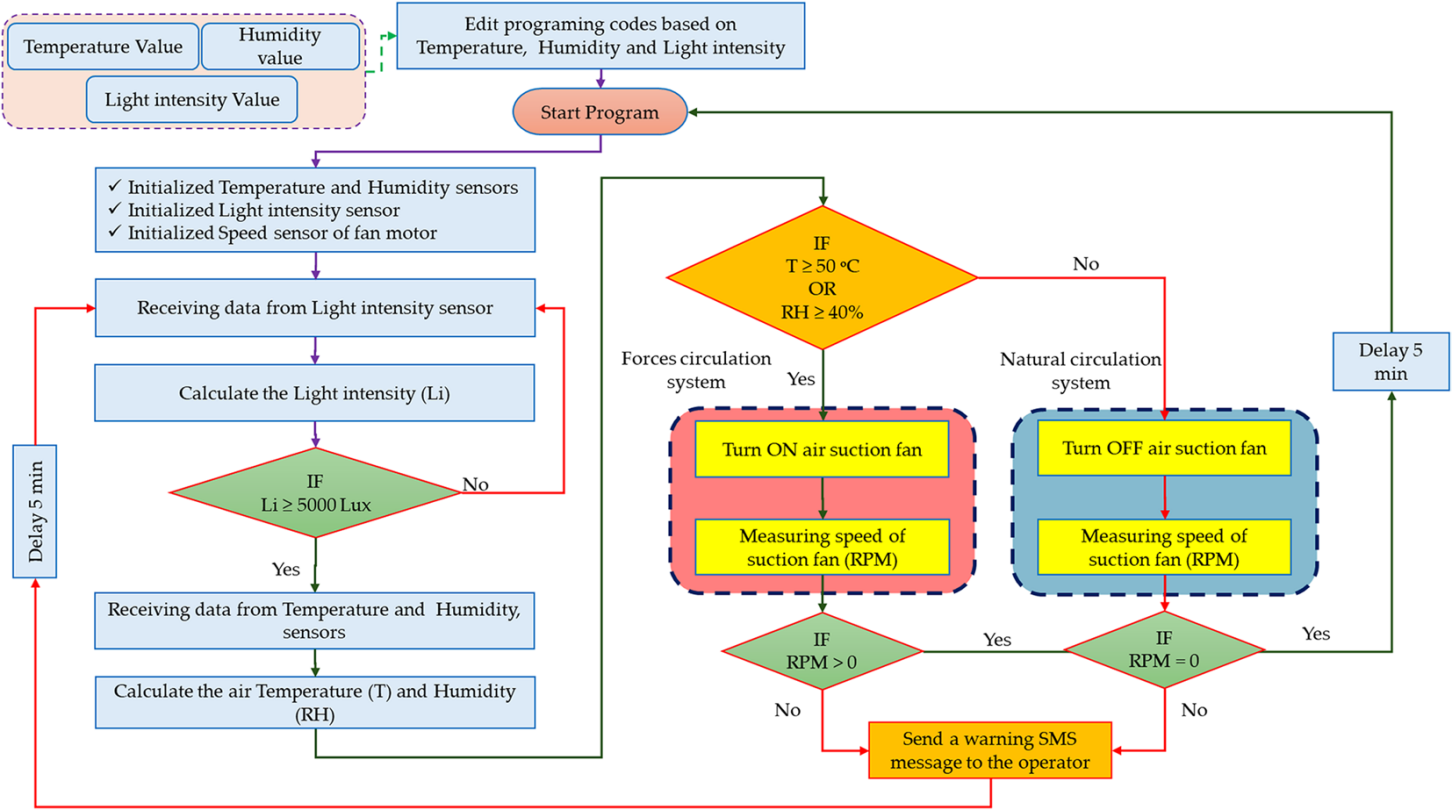
Schematic representation of the operational logic and control strategy of the developed AFNSD.

The operational algorithm of the developed AFNSD is illustrated in Fig. 3. It begins by initializing the air temperature (T), relative humidity (RH), and light intensity (Li) sensors, along with the exhaust fan motor’s speed sensor. The system first reads Li values from the environment, calculates the current intensity, and compares it to a predefined set point. If Li ≥ Set Point, the algorithm activates the exhaust fan to initiate forced ventilation. Conversely, if Li < Set Point, the fan remains off, and the algorithm collects AT and RH data. These values are then compared against their respective thresholds. When Li is low and both AT and RH are within their set points, the system operates in natural circulation mode. If Li is high or AT/RH exceed their set points, forced circulation is engaged by activating the exhaust fan. After selecting the appropriate circulation mode, the algorithm introduces a five-minute delay before restarting the cycle. The primary aim of this algorithm is to optimize the drying process by dynamically adjusting ventilation and airflow based on real-time Li, AT, and RH readings. This approach enhances drying efficiency, prevents over-drying, and reduces the risk of spoilage. The specific set points for Li, AT, and RH should be tailored to the product being dried, and further improvements could incorporate additional parameters such as the product’s MC. By automating these decisions, the AFNSD can significantly reduce energy consumption compared to conventional fossil-fuel-based dryers.

### Experimental setup

**Fig. 4:**
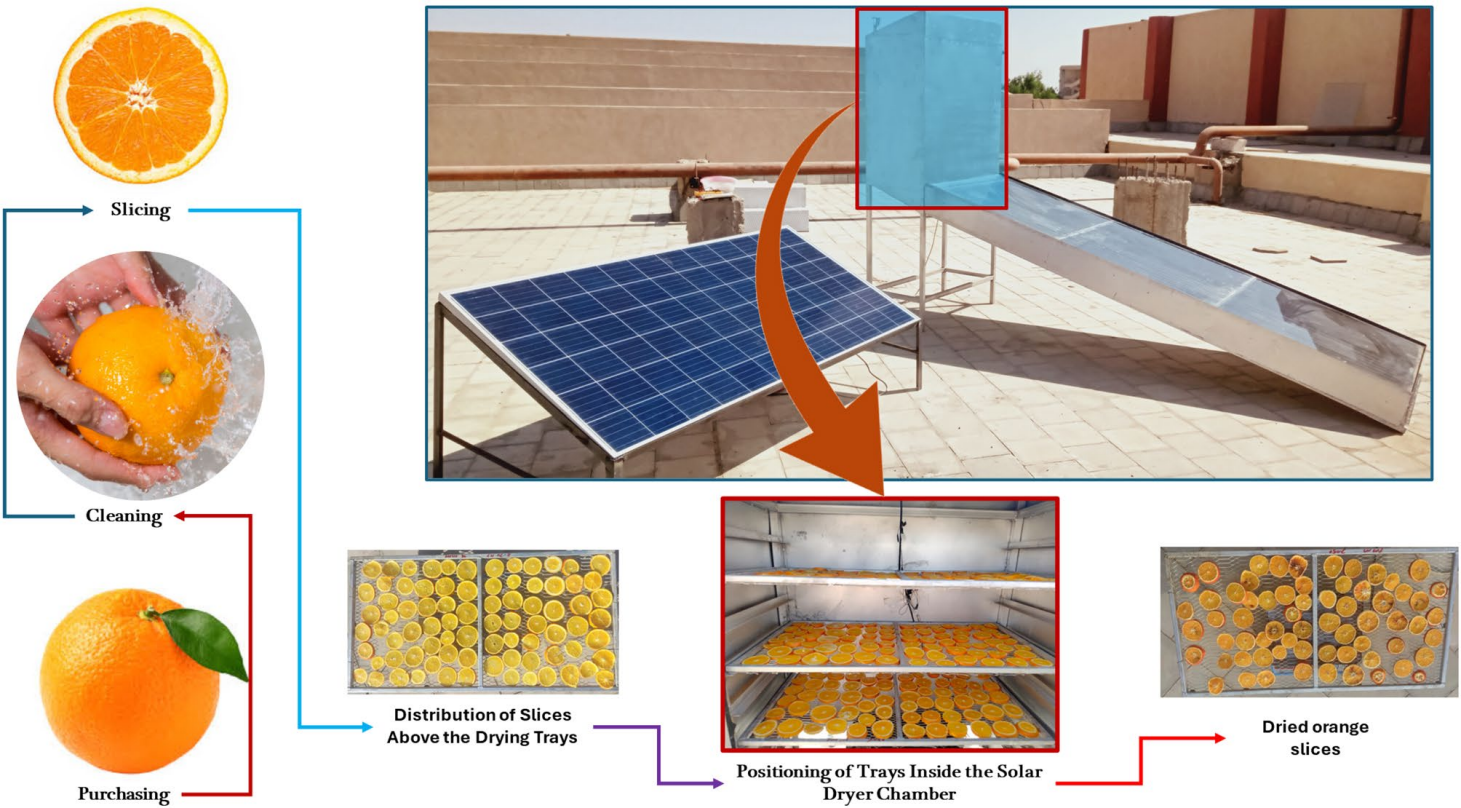
Schematic flow diagram of the sample preparation and drying procedure within the AFNSD.

During the current study, locally cultivated produce, fresh oranges, were carefully selected and purchased from a local market in Aswan, Egypt. This location was chosen due to its reliable supply of fresh fruit and proximity to the experimental site. Immediately after procurement, the oranges underwent a thorough washing process using clean tap water to eliminate any adhering dust, debris, or surface contaminants, thereby preparing them for uniform processing. Once cleaned, the oranges were manually sliced into uniform thicknesses of 4 mm, 6 mm, and 8 mm. This was done in accordance with the slicing methodology outlined by Rokhbin and Azadbakht^54^, aiming to systematically assess the impact of slice thickness on the drying kinetics and overall performance. Care was taken to maintain consistency in slice thickness to ensure the accuracy and comparability of results across all experimental runs. The sliced orange samples were then methodically arranged in a single layer across three separate drying trays to prevent overlapping, which could hinder uniform drying. The trays were vertically positioned within the DCh, each separated by an approximate distance of 20 cm. This spacing was critical to maintaining unobstructed airflow around each tray, thus optimizing heat and mass transfer during the drying process. All experimental drying trials were conducted at Aswan University in February 2025, a period characterized by intense solar irradiance typical of the region’s climatic conditions. Drying was performed using an AFNSD, a system selected for its ability to deliver controlled and consistent drying parameters. Figure 4 provides a schematic flow diagram that outlines each stage of the sample preparation and drying procedure within the AFNSD. This structured and repeatable protocol ensured experimental reliability and facilitated a comprehensive evaluation of how slice thickness and tray position influenced drying process.

### Performance analysis of the developed AFNSD

#### Drying kinetics

The fresh orange was dried at 70 °C in an electric oven until reaching constant weight. Then the initial MC was estimated using Eq. (1) ^55^.

1 $$MC~\left( {d.b.} \right)=\frac{{\mathop {\overbrace {{{{\mathrm{W}}_{\mathrm{w}}} - {\mathrm{~}}{{\mathrm{W}}_{\mathrm{d}}}}}^{{}}}\limits^{{{\mathrm{Diffrence~between~wet~and~dry~weights~of~orange~sample~}}}} }}{{\mathop {\overbrace {{{{\mathrm{W}}_{\mathrm{d}}}}}^{{}}}\limits^{{{\mathrm{Weight~of~dry~orange~sample~}}}} }} \times 100$$

The drying rate (DR) of different orange slices were calculated using Eq. (2) ^55^.

2 $$DR~\left( {{g_{water}}/{g_{dry~matter}}.h} \right)=~\frac{{Weight~loss~\left( g \right)}}{{\Delta t~\left( h \right)}}$$

#### Energy analysis

The AFNSD is compos ed of the SC and DR units. These components were analyzed based on the basic thermodynamic laws of mass and energy conservation in steady-flow systems Eqs. (3–5)^45^. As per these principles, the mass flow rate of air remains unchanged across the entire system, indicating that the rate of air entering at the inlet is exactly equal to the rate exiting at the outlet.

3 $$\sum \mathop {\overbrace {{{{\dot {m}}_{ai}}}}^{{}}}\limits^{{Inlet~mass~flow~rate}} =\sum \mathop {\overbrace {{{{\dot {m}}_{ao}}}}^{{}}}\limits^{{Outlet~mass~flow~rate}}$$

4 $$\sum \mathop {\overbrace {{{{\dot {E}}_{ai}}}}^{{}}}\limits^{{Inlet~energy~flow~rate}} =\sum \mathop {\overbrace {{{{\dot {E}}_{ao}}}}^{{}}}\limits^{{Outlet~energy~flow~rate}}$$

5 $$\mathop {\overbrace {{\dot {Q}}}^{{}}}\limits^{{{\mathrm{Heat~trnsfer~}}}} +\sum {\dot {m}_{ai}}\left( {\mathop {\overbrace {{{h_{ai}}}}^{{}}}\limits^{{{\mathrm{Inthalpy~}}}} +\frac{{\mathop {\overbrace {{{v_{ai}}}}^{{}}}\limits^{{{\mathrm{Velcity~}}}} }}{2}+\mathop {\overbrace {{{z_{ai}}}}^{{}}}\limits^{{{\mathrm{Height~}}}} g} \right)=\sum {\dot {m}_{ao}}\left( {\mathop {\overbrace {{{h_{ao}}}}^{{}}}\limits^{{{\mathrm{Inthalpy~}}}} +\frac{{\mathop {\overbrace {{{v_{ao}}}}^{{}}}\limits^{{{\mathrm{Velcity~}}}} }}{2}+\mathop {\overbrace {{{z_{ao}}}}^{{}}}\limits^{{{\mathrm{Height~}}}} g} \right)+\mathop {\overbrace {{\dot {W}}}^{{}}}\limits^{{{\mathrm{Work~done}}}}$$

Where,

6 $$\mathop {\overbrace {{\dot {W}}}^{{}}}\limits^{{{\mathrm{Work~done}}}} =zero$$

7 $$\left[ {\frac{{\mathop {\overbrace {{{v_{ai}}}}^{{}}}\limits^{{{\mathrm{Velcity~of~input~air~}}}} }}{2} - \frac{{\mathop {\overbrace {{{v_{ao}}}}^{{}}}\limits^{{{\mathrm{Velcity~of~output~air~~}}}} }}{2}} \right]\& ~\left[ {\mathop {\overbrace {{{z_{ai}}}}^{{}}}\limits^{{{\mathrm{Height~of~input~air~}}}} g - \mathop {\overbrace {{{z_{ao}}}}^{{}}}\limits^{{{\mathrm{Height~of~output~air~~}}}} g} \right]=~{\mathrm{very~small~and~it~is~neglected}}$$

Equations (8) and (9) were obtained for the SC form Eqs. (3) and (4).

8 $$\sum \mathop {\overbrace {{{{\dot {m}}_{ai}}}}^{{}}}\limits^{{Inlet~mass~flow~rate}} =\sum \mathop {\overbrace {{{{\dot {m}}_{ao}}}}^{{}}}\limits^{{Outlet~mass~flow~rate}} =\sum \mathop {\overbrace {{{{\dot {m}}_a}}}^{{}}}\limits^{{Mass~flow~rate}}$$

9 $$\mathop {\overbrace {{\dot {Q}}}^{{}}}\limits^{{{\mathrm{Heat~trnsfer~}}}} =\mathop {\overbrace {{{{\dot {Q}}_{u,~~{\mathrm{SC}}}}}}^{{}}}\limits^{{{\mathrm{Useful~energy~~}}}} =\mathop {\overbrace {{{{\dot {Q}}_{in,~~{\mathrm{SC}}}}}}^{{}}}\limits^{{{\mathrm{Input~energy~}}}} - \mathop {\overbrace {{{{\dot {Q}}_{ls,~~{\mathrm{SC}}}}}}^{{}}}\limits^{{{\mathrm{Energy~loss}}}} =\mathop {\overbrace {{{{\dot {m}}_a}}}^{{}}}\limits^{{Air~mass~flow~rate}} \left( {\mathop {\overbrace {{{h_{ao}} - {h_{ai}}}}^{{}}}\limits^{{Change~in~enthalpy}} } \right)$$

Where the ${\dot {Q}_{in,~~{\mathrm{SC}}}}$, ${\dot {Q}_{u,~~{\mathrm{SC}}}}$ and ${\eta _{en,~~SC}}$ of the SC were calculated according to Eqs. (10–12)^56–58^.

10 $$\mathop {\overbrace {{{{\dot {Q}}_{in,~~{\mathrm{SC}}}}}}^{{}}}\limits^{{{\mathrm{Input~energy~}}}} =~\mathop {\overbrace {{{I_s}}}^{{}}}\limits^{{{\mathrm{Solar~radation~intensity~}}}} \times \mathop {\overbrace {{{A_{SC}}}}^{{}}}\limits^{{{\mathrm{Surface~area~of~the~solar~collector~}}}}$$

11 $$\overbrace {{{{\dot {Q}}_{u,~~{\mathrm{SAC}}}}}}^{{{\mathrm{Useful~energy}}}}=~\mathop {\overbrace {{{{\dot {m}}_a}}}^{{}}}\limits^{{Mass~flow~rate}} \times \mathop {\overbrace {{{C_{pa}}}}^{{}}}\limits^{{{\mathrm{Specific~heat~of~air}}}} \times \left( {\mathop {\overbrace {{{T_{co}} - {T_{ci}}}}^{{}}}\limits^{{Change~in~air~temperature}} } \right)$$

12 $$\mathop {\overbrace {{{\eta _{e,~~SC}}}}^{{}}}\limits^{{{\mathrm{Energy~efficiency~}}}} =\frac{{{{\dot {Q}}_{u,~~{\mathrm{SC}}}}}}{{{{\dot {Q}}_{in,~~{\mathrm{SC}}}}}}=\frac{{{{\dot {m}}_a}{C_{pa}}\left( {{T_{co}} - {T_{ci}}} \right)}}{{{I_s}{A_{SC}}}}$$

#### Exergy analysis ($\mathop {Ex}\limits^{.}$)

Exergy ($\mathop {Ex}\limits^{.}$) represents the usable portion of energy ($\:\dot{Q}$) within the AFNSD and serves as a measure of energy quality. The analysis of $\mathop {Ex}\limits^{.}$ for the AFNSD is grounded in the second law of thermodynamics and is determined using Eq. (13).

13 $$\begin{aligned} \mathop {\overbrace {{\mathop {Ex}\limits^{.} }}^{{}}}\limits^{{{\mathrm{Exergy~}}}} & =\left( {\mathop {\overbrace {{u - {u_\infty }}}^{{}}}\limits^{{{\mathrm{Internal~energy}}}} } \right) - {T_0}\left( {\mathop {\overbrace {{s - {s_\infty }}}^{{}}}\limits^{{E{\mathrm{ntropy}}}} } \right)+{P_0}\left( {\mathop {\overbrace {{v - {v_\infty }}}^{{}}}\limits^{{F{\mathrm{low~work}}}} } \right)+\mathop {\overbrace {{\frac{{{V^2}}}{2}}}^{{}}}\limits^{{M{\mathrm{omentum~energy}}}} +g\left( {\mathop {\overbrace {{z - {z_\infty }}}^{{}}}\limits^{{G{\mathrm{ravitational~energy}}}} } \right) \\ & \;\;\;+\mathop \sum \limits_{{ch}} \left( {\mathop {\overbrace {{{\mu _{ch}} - {\mu _\infty }}}^{{}}}\limits^{{{\mathrm{Chemical~energy~}}}} } \right) \times {N_{ch}}+\left( {\mathop {\overbrace {{\sigma {A_i}{F_i}\left( {3{T^4} - T_{\infty }^{4} - 4{T_\infty }{T^3}} \right)}}^{{}}}\limits^{{R{\mathrm{adiation~energy}}}} } \right) \\ \end{aligned}$$

Equation (14) was derived from Eq. (13) by simplifying it—specifically, by omitting terms that were not relevant to the drying process, resulting in a more concise and applicable form^59^.

14 $$\mathop {Ex}\limits^{.} =~{\dot {m}_a}{C_{pa}}\left( {{T_0}ln\left( {\frac{T}{{{T_0}}}} \right)} \right)$$

where, ${T_0}$ is atmospheric temperature.

##### Exergy analysis of the SC

$\mathop {Ex}\limits^{.}$ balance for the SC is given by Eqs. (15–18)^58,60,61^,

15 $$\overbrace {{{{\mathop {Ex}\limits^{.} }_{ls,~SC}}}}^{{{\mathrm{Exergy~loss}}}}=\overbrace {{{{\mathop {Ex}\limits^{.} }_{in,~SC}}}}^{{{\mathrm{Input~exergy}}}} - \overbrace {{{{\mathop {Ex}\limits^{.} }_{out,~SC}}}}^{{{\mathrm{Output~exergy}}}}$$

16 $${\mathop {Ex}\limits^{.} _{in,~SC}}=\left[ {1 - \frac{{{T_0}}}{{\mathop {\overbrace {{{T_s}}}^{{}}}\limits^{{{\mathrm{Sun~temperature~}}\left( {6000{\mathrm{~k}}} \right){\mathrm{~}}}} }}} \right] \times \mathop {\overbrace {{{{\dot {Q}}_{in,~abs}}}}^{{}}}\limits^{{{\mathrm{Energy~absorbed~by~the~absorper~plate~}}}}$$

17 $${\dot {Q}_{in,~abs}}=\mathop {\overbrace {\tau }^{{}}}\limits^{{{\mathrm{Transmissivity~of~~glass~}}\left( {0.88} \right){\mathrm{~}}}} ~ \times \mathop {\overbrace {\alpha }^{{}}}\limits^{{{\mathrm{Absorptivity~of~~glass~}}\left( {0.95} \right){\mathrm{~}}}} ~ \times {\dot {Q}_{in,~~{\mathrm{SC}}}}$$

18 $${\mathop {Ex}\limits^{.} _{out,~SC}}={\dot {m}_a}{C_{pa}}\left( {\left( {{T_{co}} - {T_{ci}}} \right) - {T_0}ln\left( {\frac{{{T_{co}}}}{{{T_{ci}}}}} \right)} \right)$$

19 $$~\mathop {\overbrace {{{\eta _{ex,~~SC}}}}^{{}}}\limits^{{{\mathrm{Exergy~efficiency~}}}} =\frac{{{{\mathop {Ex}\limits^{.} }_{out,~SC}}}}{{{{\mathop {Ex}\limits^{.} }_{in,~SC}}}}=1 - \frac{{{{\mathop {Ex}\limits^{.} }_{ls,~SC}}}}{{{{\mathop {Ex}\limits^{.} }_{in,~SC}}}}=1 - \frac{{{T_0}{S_{gen}}}}{{\left[ {1 - \frac{{{T_0}}}{{{T_s}}}} \right]{{\dot {Q}}_{in,~~SC}}}}$$

##### $\mathop {Ex}\limits^{.}$ analysis of the DCh

$\mathop {Ex}\limits^{.}$ balance for the DCh is formulated to account for all exergy inputs, outputs, and losses within the system (Eqs. 20–23). This balance provides a comprehensive understanding of the exergy behavior and efficiency of the drying process. It is mathematically represented as follows:

20 $$\overbrace {{{{\mathop {Ex}\limits^{.} }_{ls,~DCh}}}}^{{Exergy~loss}}=\overbrace {{{{\mathop {Ex}\limits^{.} }_{in,~DCh}}}}^{{Input~exergy}} - \overbrace {{{{\mathop {Ex}\limits^{.} }_{out,DCh}}}}^{{Output~exergy}}$$

21 $$\mathop {Ex}\limits^{.}_{in, DCh} = \dot{m}_{a} C_{pa} \left( {\left( {T_{in,DCh} - T_{0} } \right) - T_{0} ln\left( {\frac{{T_{in,DCh} }}{{T_{0} }}} \right)} \right)$$

22 $$\mathop {Ex}\limits^{.}_{out, DCh} = \dot{m}_{a} C_{pa} \left( {\left( {T_{out,DCh} - T_{ci} } \right) - T_{0} ln\left( {\frac{{T_{out,DCh} }}{{T_{0} }}} \right)} \right)$$

23 $$\eta_{ex, DCh} = \frac{{\mathop {Ex}\limits_{out, DCh} }}{{\mathop {Ex}\limits_{in, DCh} }}$$

#### Sustainability indicators

This study used three exergy-based sustainability indicators—IP, WER, and SI—to evaluate the AFNSD’s performance. These indicators assess exergy input, losses, and system efficiency. As exergy losses increase, IP and WER rise, while SI declines. Their mathematical formulations are given below^59^.

24 $$IP = \left( {1 - \eta_{ex} } \right)\mathop {Ex}\limits_{ls}$$

25 $$WER = \frac{{\mathop {Ex}\limits_{ls} }}{{\mathop {Ex}\limits_{in} }}$$

26 $$SI=~\frac{1}{{1 - {\eta _{ex}}}}$$

### Uncertainty analysis

The measurement uncertainties for temperature, relative humidity, wind speed, and solar radiation were found to be 0.34%, 0.29%, 0.23%, and 0.14%, respectively. Taking all these variables into account, the overall uncertainty in evaluating the efficiency of the SD was estimated at around ± 2%.

27 $${\mathcal{W}_r}={\left[ {{{\left( {\frac{{\partial R}}{{\partial {x_1}}}{\mathcal{W}_1}} \right)}^2}+{{\left( {\frac{{\partial R}}{{\partial {x_2}}}{\mathcal{W}_2}} \right)}^2}+ \ldots +{{\left( {\frac{{\partial R}}{{\partial {x_3}}}{\mathcal{W}_3}} \right)}^2}} \right]^{1/2}}$$

## Results and discussion

**Fig. 5:**
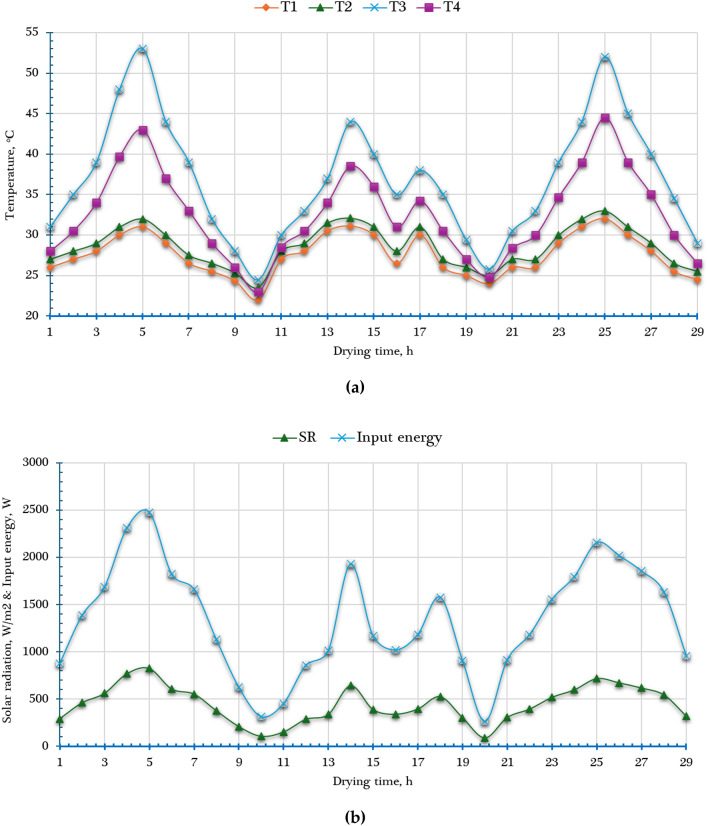
Weather conditions during field experiments. Whereas (a) temperature, and (b) solar radiation and input energy.

The drying experiments involving fresh orange slices were conducted in January 2025 at Aswan University, Egypt. The primary objective of the study was to evaluate how varying slice thicknesses—specifically 4 mm, 6 mm, and 8 mm—and the vertical positioning of trays within the AFNSD (categorized as lower, middle, and upper levels) influence drying performance. To ensure the reliability and reproducibility of the results, each experimental condition was replicated three times. Laboratory measurements determined that the fresh orange slices had an average initial moisture content of 5.94 g water/g dry matter. During the course of the experiments, environmental conditions fluctuated. Ambient air temperatures in shaded areas ranged from 22 °C to 32 °C, while solar radiation levels varied widely between 88 and 826 W/m^2^. Natural wind speeds also showed variability, ranging from 0.1 to 0.4 m/s. Inside the AFNSD, the temperature of the drying air was observed to vary from 24.6 °C to 49.2 °C, reflecting the influence of solar energy and internal airflow regulation within the system. To illustrate these variations, Fig. 5 presents the average solar radiation intensity along with temperature profiles both inside and outside the AFNSD throughout the drying process, offering a clear view of the system’s thermal behavior. All experiments were carried out under a uniform airflow rate of 0.13 m^3^/s, maintaining consistent drying conditions across all treatments. This well-controlled experimental setup enabled an accurate and comprehensive analysis of the impact of slice thickness and tray placement on the drying kinetics and performance of orange slices.

### Drying kinetics

**Fig. 6:**
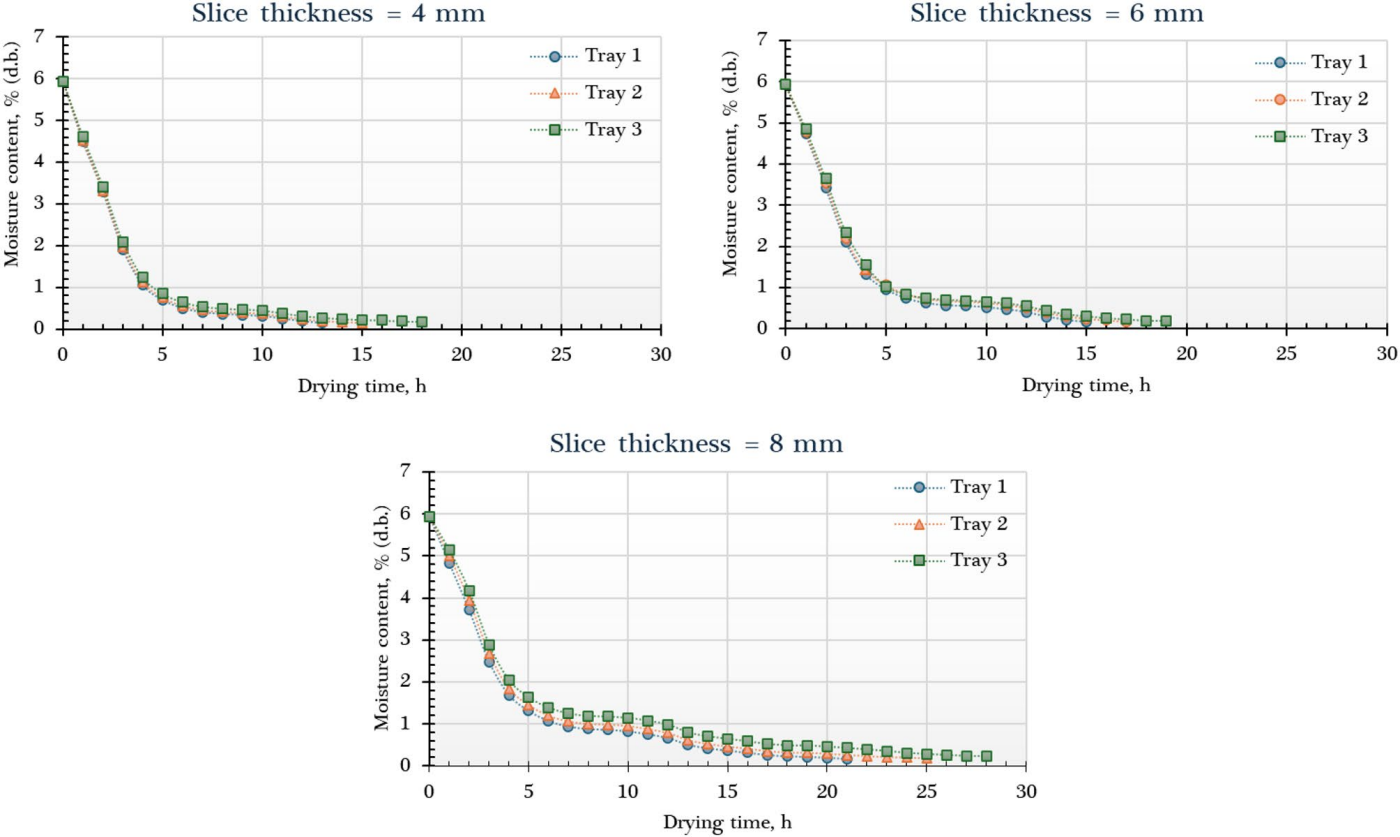
Moisture content of different orange samples dried using the AFNSD.

Figure 6 displays the moisture content of dried orange slices processed in the AFNSD, analyzed by tray position and slice thickness. The initial weights of the slices differed based on thickness and tray placement. For 4 mm slices, the lower, middle, and upper trays held 1890 g, 1920 g, and 1910 g, respectively. At 6 mm thickness, the weights were 2815 g (lower), 2820 g (middle), and 2810 g (upper). The 8 mm slices weighed 3800 g (lower), 2820 g (middle), and 3985 g (upper). These differences arise from variations in slice thickness and tray loading, affecting drying dynamics. The initial average MC of the oranges was approximately 5.94 g _water_/g _dry matter_ (d.b.). Drying times to reach the target MC ranged from 13 to 25 h, influenced by slice thickness and tray position. Thinner slices (4 mm) on lower trays dried fastest, while thicker slices (8 mm) on upper trays took the longest. This aligns with expectations, as greater thickness increases the moisture diffusion path, prolonging drying and yielding higher final MC. The lowest final MC (0.14 g g _water_/g _dry matter_) occurred with 4 mm slices on the lower tray, where temperatures were highest, compared to 0.16 and 0.17 g _water_/g _dry matter_ for 6 mm and 8 mm slices, respectively. Thinner slices facilitated faster moisture loss, reducing drying time and final MC. These results agree with prior research^62–69^. Furthermore, studies on multi-tray dryers indicate that trays in warmer or better-ventilated zones (typically near the air inlet) dry more efficiently and achieve lower final MC than those in less optimal positions^66,67,70^.

**Fig. 7:**
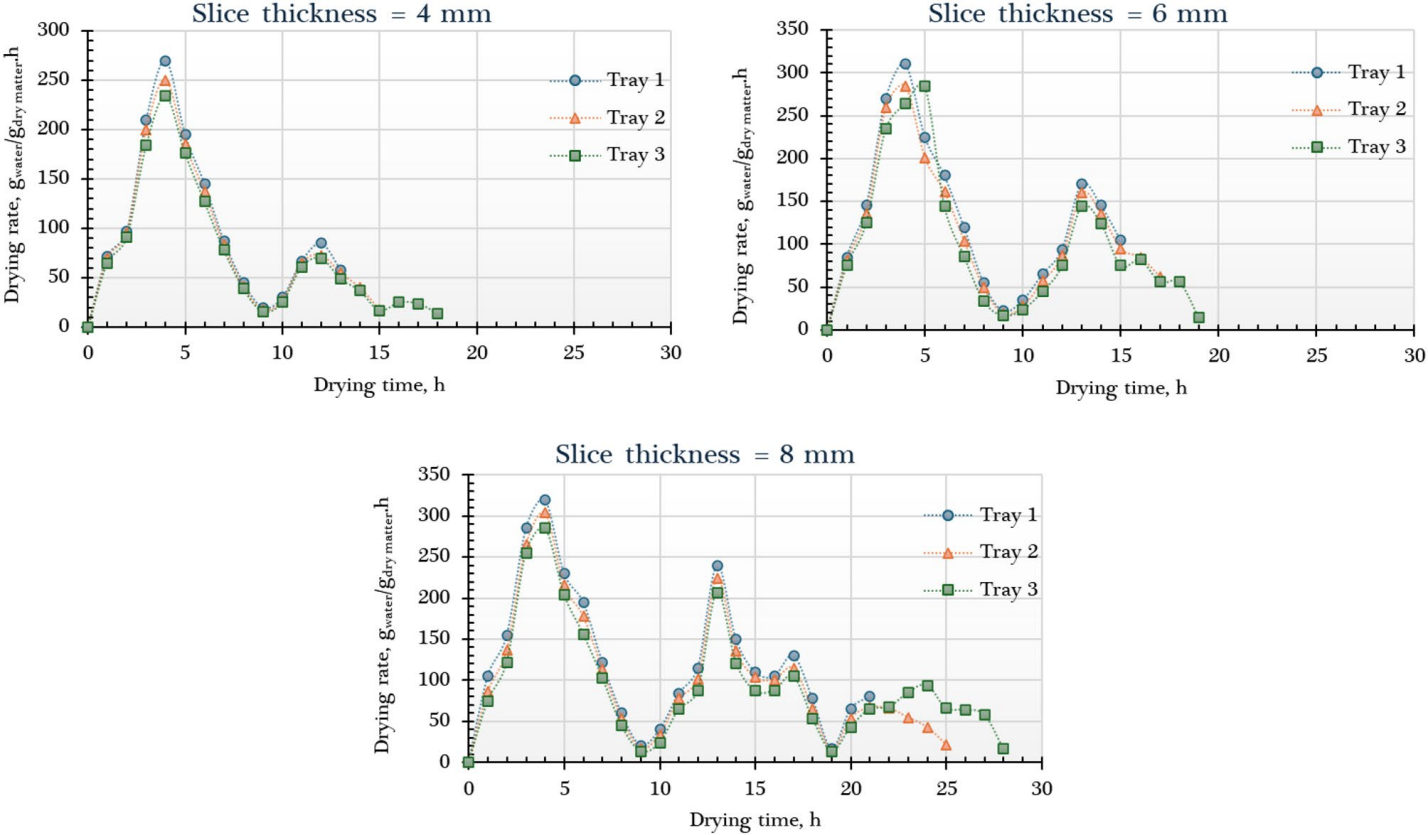
Drying rate of different orange samples dried using the AFNSD.

Figure 7 presents the DR curves of orange slices at varying tray positions and thicknesses. The results indicate that the highest DR was achieved in samples on the lower trays, surpassing those on the middle and upper trays. This difference stems from the elevated temperatures at the lower level, where direct contact with incoming hot air from the solar collector intensifies drying. For example, with 4 mm slices, the DR values were approximately 270, 250, and 234 g _water_/g _dry matter_/h for the lower, middle, and upper trays, respectively. Regarding thickness, the DR rose with increasing slice size, reaching about 270, 310, and 320 g _water_/g _dry matter_/h for 4, 6, and 8 mm slices, respectively, on the lower trays. This trend may be linked to the higher initial moisture content and greater sample mass in thicker slices, which boosted evaporation rates early in the drying process. In summary, both tray placement and slice thickness significantly influenced the DR, with the most efficient drying observed in thicker slices positioned on the lower trays.

### Energy analysis of the SC

**Fig. 8:**
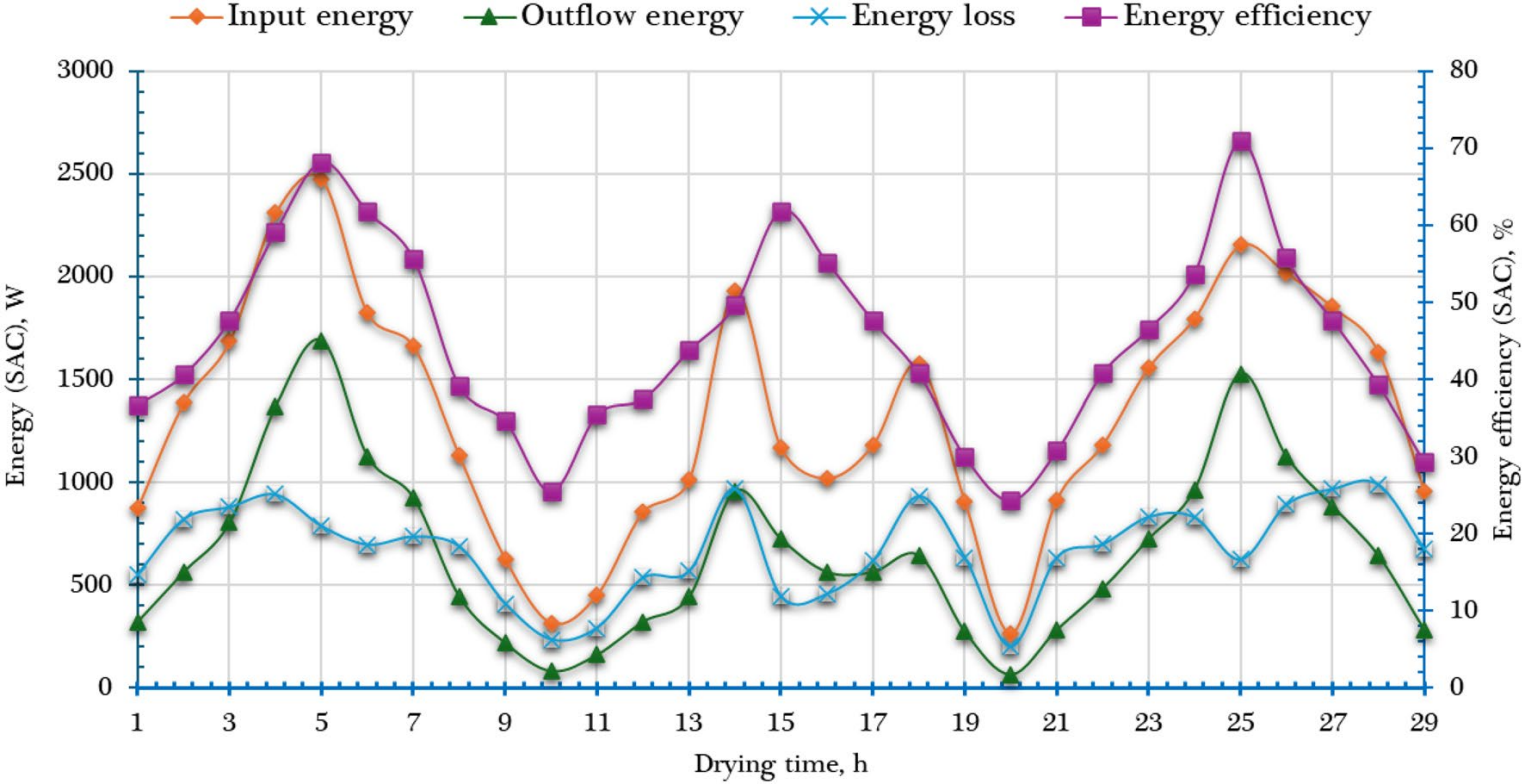
Energy analysis of the SC (${\dot {Q}_{in,~~{\mathrm{SC}}}}$, ${\dot {Q}_{u,~~{\mathrm{SC}}}}$, ${\dot {Q}_{ls,~~{\mathrm{SC}}}}$, and ${\eta _{en,~~SC}}$).

Figure 8 presents the hourly energy performance analysis of the SC within the AFNSD system. This analysis includes four key parameters: the total input energy (${\dot {Q}_{in,~~{\mathrm{SC}}}}$), useful energy (${\dot {Q}_{u,~~{\mathrm{SC}}}}$), loss energy (${\dot {Q}_{ls,~~{\mathrm{SC}}}}$), and efficiency (${\eta _{en,~~SC}}$). These were determined on an hourly basis by taking into account solar radiation intensity and the temperature difference between the air entering and exiting the solar collector. The input energy ${\dot {Q}_{in,~~{\mathrm{SC}}}}$​ represents the total solar energy incident on the SC surface. It depends on several factors, including solar irradiance, collector surface area, and its orientation with respect to the sun. During the experiment, the total solar energy input ranged from 264 W to 2478 W throughout the day. The useful energy gain ${\dot {Q}_{u,~~SC}}$​, as shown in Fig. 8 and estimated using Eq. (11), reflects the portion of solar energy effectively used to heat the air within the collector. This value varied between 64.4 W and 1689.8 W, largely influenced by the level of incident solar radiation. The relatively higher ${\dot {Q}_{u,~~SC}}$​​ values were attributed to the continuous operation of exhaust fans, which enhanced air movement and heat transfer within the system. The energy efficiency ${\eta _{en,~~SC}}$​ was calculated using Eq. (12) and is also illustrated in Fig. 8. Since efficiency is directly linked to the ratio of useful to input energy, it followed a diurnal pattern—rising steadily through the morning hours and reaching a peak around midday, then declining by late afternoon. The efficiency ranged between 24.38% and 70.98%, with the highest value recorded at 12:00 p.m. due to maximum solar intensity at that time. Table 1 provides a comparative assessment of the obtained ${\eta _{en,~~SC}}$​ with those reported in previous studies, demonstrating the performance of the developed system relative to established solar drying technologies.

**Table 1:** Comparing the obtained ${\eta _{en,~~SC}}$ with different types of SCs.

Ref.	Type	${\boldsymbol{\eta}_{{\boldsymbol{e}}{\boldsymbol{n}},~~{\boldsymbol{S}}{\boldsymbol{C}}}}$, %
^71^	Natural and forced SC	62%
^72^	Multi-pass SC	52.1%
^73^	Top and bottom flow SC	50.0%
^58^	Solar tunnel dryer	27.45%–42.50%
^74^	Flat plate SC	45.32%
Current study	AFNSD	24.38–70.98%

### Exergy analysis ($\mathop {Ex}\limits^{.}$)

#### Exergy analysis of the SC ($\mathop {Ex}\limits^{.} _{{SC}}$)

**Fig. 9:**
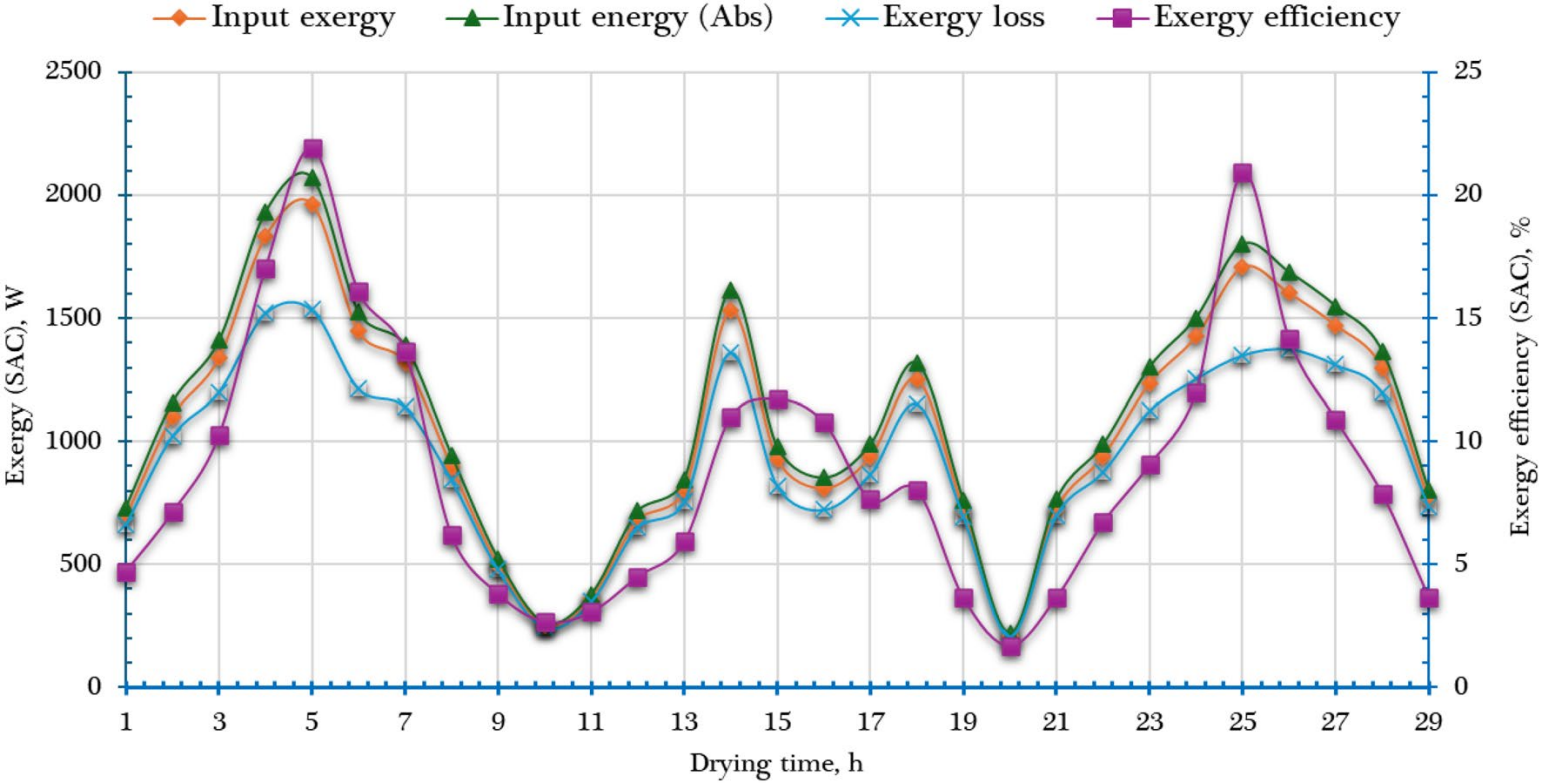
Exergy analysis of the SC ($\mathop {Ex}\limits^{.}_{in, SC}$, $\mathop {Ex}\limits^{.}_{out, SC}$, $\mathop {Ex}\limits^{.}_{ls, SC}$ and ${\eta _{ex,~~SC}}$).

Figure 9 illustrates the hourly variation in exergy performance parameters of the SC, including inlet exergy ($\mathop {Ex}\limits^{.}_{in, SC}$), outlet exergy ($\mathop {Ex}\limits^{.}_{out, SC}$), exergy loss ($\mathop {Ex}\limits^{.}_{ls, SC}$), and exergy efficiency (${\eta _{ex,~~SC}}$), all calculated using Eqs. (11–13). The exergy input $\mathop {Ex}\limits^{.}_{in, SC}$, output $\mathop {Ex}\limits^{.}_{out, SC}$, and loss $\mathop {Ex}\limits^{.}_{ls, SC}$ are closely linked to the intensity of incident solar radiation. As shown in Fig. 9, these values increased steadily from early morning to midday—when solar radiation was strongest—and then declined gradually in the afternoon, following the typical solar radiation curve. The $\mathop {Ex}\limits^{.}_{in, SC}$was computed using Eq. (10), which considers solar radiation and ambient temperature. During the experimental period, $\mathop {Ex}\limits^{.}_{in, SC}$​ ranged from 220 W in the early morning to a maximum of 2071.61 W at 12:00 p.m., corresponding with peak solar irradiance. The $\mathop {Ex}\limits^{.}_{out, SC}$, representing the useful exergy transferred to the air within the SC, varied from 3.54 W to 431.20 W throughout the day. Similarly, the exergy loss $\mathop {Ex}\limits^{.}_{ls, SC}$—the portion of exergy destroyed due to irreversibilities—ranged from 206.16 W to 1534.76 W, with the highest losses also occurring at noon due to the elevated radiation levels. Average values calculated over the drying period were: 1116.8 W ($\mathop {Ex}\limits^{.}_{in, SC}$), 117 W ($\mathop {Ex}\limits^{.}_{out, SC}$), and 943.4 W ($\mathop {Ex}\limits^{.}_{ls, SC}$). The ${\eta _{ex,~~SC}}$, determined using Eq. (12), reflects the ratio of useful exergy output to the total exergy input. As shown in Fig. 9, it ranged from 1.69% to 21.93%, with an average value of approximately 9%. Notably, while exergy input primarily depends on solar radiation, the exergy output is more influenced by the temperature of the outlet air. Since solar radiation remains relatively constant across similar conditions, variations in ${\eta _{ex,~~SC}}$​ are largely governed by changes in outlet air temperature. These findings highlight the SC’s performance characteristics and emphasize the role of thermal and solar conditions in influencing its exergy behavior. Table 2 provides a comparative assessment of the obtained ${\eta _{ex,~~SC}}$​ with those reported in previous studies.

**Table 2:** Comparing the obtained ${\eta _{ex,~~SC}}$ with different types of SCs.

Ref.	Type	${\boldsymbol{\eta}_{{\boldsymbol{e}}{\boldsymbol{x}},~~{\boldsymbol{S}}{\boldsymbol{C}}}}$
^75^	Forced convection indirect SD	2.44%
	Natural convection indirect SD	2.03%
^76^	Active greenhouse dryer	3.45%
^58^	Solar tunnel dryer	41.42%
^77^	Mixed-mode SD	18.8–41.4%
^78^	Hybrid mixed mode greenhouse SD	19.11–28.96%
^58^	Solar tunnel dryer	32–69%
^74^	Indirect type natural convection SD	7.4-45.23%
Current study	AFNSD	1.69–21.93%

#### Exergy analysis of the DCh ($\mathop {Ex}\limits^{.}_{DCh}$)

**Fig. 10:**
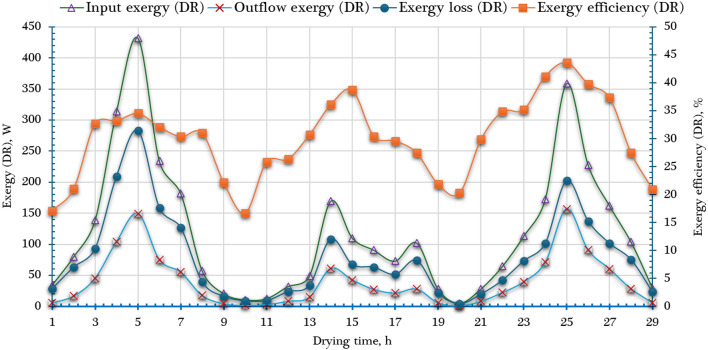
Exergy analysis of the DCh ($\mathop {Ex}\limits^{.}_{in, DR}$, $\mathop {Ex}\limits^{.}_{out,DR}$, $\mathop {Ex}\limits^{.}_{ls, DR}$ and ${\eta _{ex,~~DR}}$).

The inlet exergy ($\mathop {Ex}\limits^{.}_{in, DCh}$​), outlet exergy ($\mathop {Ex}\limits^{.}_{out,DCh}$​), and exergy loss ($\mathop {Ex}\limits^{.}_{ls, DCh}$​) for the DCh were determined based on Eqs. (20) to (23), and the results are presented in Fig. 10. Notably, the inlet exergy to the DCh is primarily influenced by the temperature of the air exiting the SC, as it directly affects the thermal energy carried into the DCh. According to the data illustrated in Fig. 10, $\mathop {Ex}\limits^{.}_{in, DCh}$ ranged between 3.93 W and 586.49 W, while $\mathop {Ex}\limits^{.}_{out,DCh}$ varied from 0.65 W to 355.97 W. The exergy losses, represented by $\mathop {Ex}\limits^{.}_{ls, DCh}$​, fell within the range of 4.12 W to 283.09 W. These values exhibit a pronounced increase during midday, which coincides with the highest inlet air temperatures to the drying chamber, thus enhancing the thermodynamic potential of the air stream. The trends observed for these exergy parameters are primarily governed by two key variables: the temperature and mass flow rate of the drying air. In the developed AFNSD, the inlet air temperature to the DCh remains within a moderate range of approximately 24.5 °C to 53 °C. However, the system features a relatively high mass flow rate, around 0.13 m³/s. This higher airflow significantly contributes to greater exergy transfer rates, particularly around midday when thermal conditions are most favorable. As shown in Fig. 10, the ${\eta _{ex,~~DCh}}$, calculated using Eq. (23), varies over the course of the drying day. The ${\eta _{ex,~~DCh}}$ ranges from 16.70% to 43.64%, reflecting the proportion of useful exergy (i.e., outlet exergy) relative to the exergy input. This efficiency tends to increase with drying time, which can be attributed to the progressively smaller temperature differential between the DCh inlet and outlet. As drying proceeds, the moisture content of the product diminishes, resulting in a reduced heat demand and a smaller drop in air temperature across the chamber. Consequently, more of the input exergy is retained at the outlet, leading to an apparent improvement in ${\eta _{ex,~~DCh}}$​ as the drying session progresses. Table 3 shows the comparison between the obtained ${\eta _{ex,~~DCh}}$ with different types of solar dryers.

**Table 3:** Comparing the obtained ${\eta _{ex,~~DCh}}$ with different types of solar dryers.

Refs.	Type	${\boldsymbol{\eta}_{{\boldsymbol{e}}{\boldsymbol{x}},~~{\boldsymbol{D}}{\boldsymbol{C}}{\boldsymbol{h}}}}$
^75^	Forced convection indirect solar dryer	16.19–97.75%
	Natural convection indirect solar dryer	15.17–91.08%
^56^	Natural convection SD	55.35–79.35%
^79^	Triple-pass SD	2.8-87.02%
^58^	Solar tunnel dryer	41.42%
^80^	Mixed mode forced convection solar tunnel dryer	23.25–73.31%
Current study	AFNSD	16.7 and 43.64%

### Sustainable indicators

To comprehensively assess the thermodynamic performance and environmental sustainability of the developed AFNSD, key exergy-based sustainability indicators—namely the IP, WER, and SI—were calculated. These indicators were used to evaluate the [$\mathop {Ex}\limits^{.}_{ls, DCh}$& $\mathop {Ex}\limits^{.}_{ls, SC}$] and [${\eta _{ex,~~DCh}}$ & ${\eta _{ex,~~SC}}$] in relation to the total [$\mathop {Ex}\limits^{.}_{in, DCh}$ & $\mathop {Ex}\limits^{.}_{in, SC}$].

The application of these indicators is critical not only for performance analysis but also for guiding future improvements in the design and operation of the DCh and SC. By analyzing IP, WER, and SI, researchers and engineers can identify thermodynamic inefficiencies, reduce unnecessary exergy destruction, and enhance the overall energy sustainability of the drying process. This approach ensures that the drying system operates more efficiently while minimizing its environmental impact.

The IP, which indicates the possible enhancement in system performance if all irreversibilities were eliminated, was found to vary from 2.03 to 12.61 W in the SC and from 0.03 to 1.85 W in the DCh. These values highlight that the SC has a larger potential for efficiency improvement compared to the DCh.

**Fig. 11:**
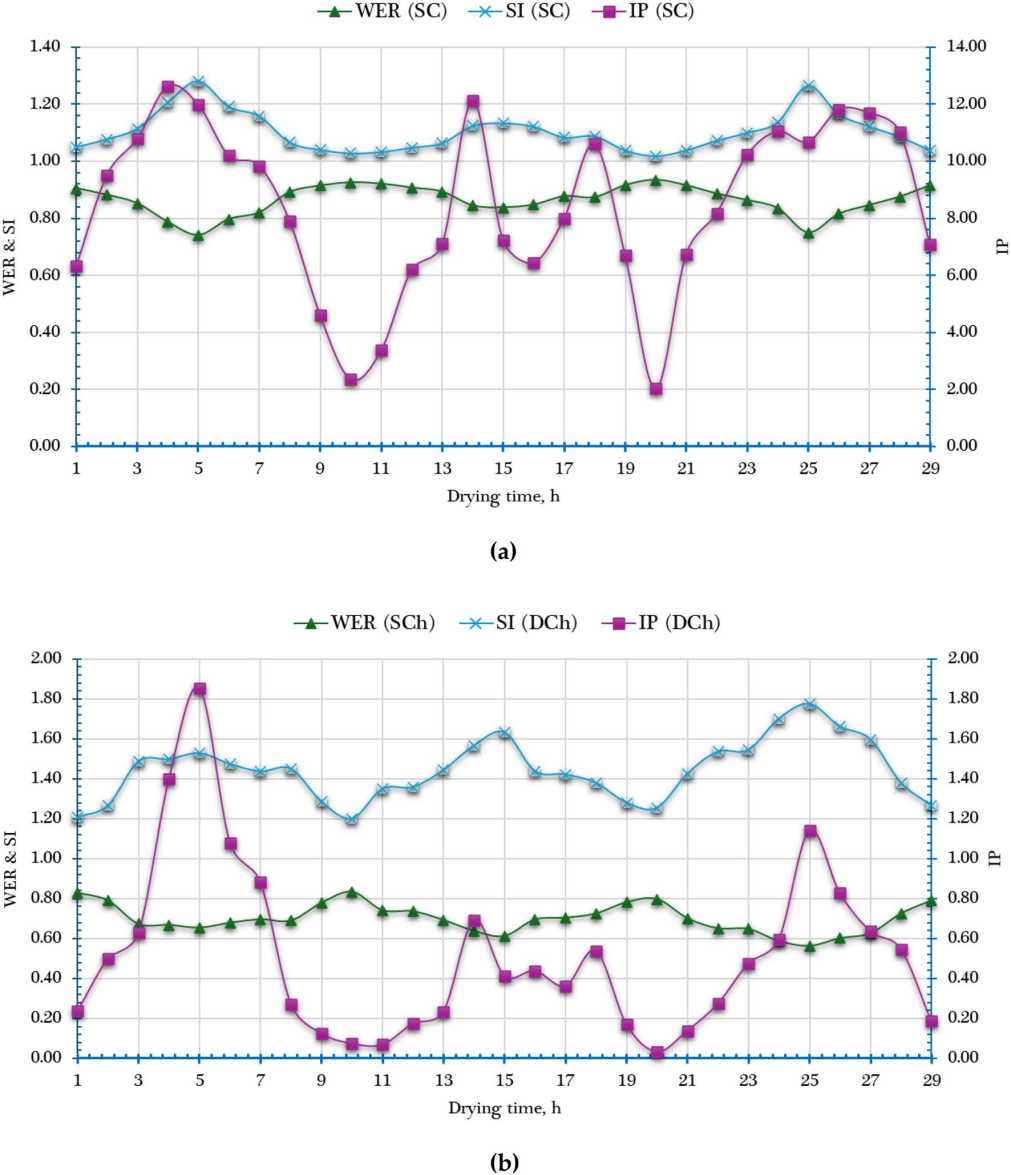
Sustainability indicators of both SC and DCh.

Furthermore, the WER and SI were computed using Eqs. (25) and (26), and the results are presented in Fig. 11. WER, which quantifies the proportion of input exergy that is wasted, showed a decreasing trend with increasing temperature. The lowest WER values were recorded around midday (approximately 12:00 p.m.), corresponding to peak thermal conditions. The average WER values were 0.9 for the SC and 0.7 for the DCh, indicating moderate exergy waste levels, especially in the SC.

In contrast, the SI—a measure of system sustainability based on the balance between useful output and wasted exergy—ranged from 1.02 to 1.28 in the SC and from 1.2 to 1.77 in the DCh. These values suggest that the DCh exhibited slightly better sustainability performance compared to the SC, likely due to more efficient heat utilization and lower relative exergy destruction.

## Conclusions

This study successfully integrated electronic control systems—comprising sensors and a microcontroller—into the Active Forced–Natural Solar Dryer (AFNSD) to enable automatic regulation of temperature, humidity, and airflow. This automation eliminated the need for manual intervention, reduced energy losses, and maintained stable drying conditions, thereby improving process efficiency and consistency.

Thermodynamic analyses demonstrated that the AFNSD achieved competitive performance in terms of both energy and exergy efficiencies, while also yielding favorable sustainability indicators. The solar collector’s energy efficiency ranged from 24.38% to 70.98%, with a peak at midday, indicating effective utilization of available solar energy. Although exergy efficiencies were lower—between 1.69% and 21.93% for the solar collector and 16.7% to 43.64% for the drying chamber—these values are consistent with expected thermodynamic limitations and highlight areas where heat losses could be minimized to improve system performance.

The drying trials with orange slices revealed that both slice thickness and tray position had a marked influence on drying kinetics. Thinner slices (4 mm) placed on lower trays dried significantly faster, requiring as little as 13 h to reach the target moisture content, while thicker slices (8 mm) on upper trays required up to 25 h. These findings underscore the importance of optimizing product loading and arrangement to shorten drying times and enhance throughput.

Sustainability metrics, including IP, WER, and SI, indicated that the AFNSD operates with relatively low irreversibilities and a modest environmental footprint. For instance, the average WER was 0.9 for the solar collector and 0.7 for the drying chamber, while the SI values suggested stable and sustainable operation under varying solar radiation conditions.

Overall, the integration of automated control not only improved the precision of drying conditions but also contributed to better thermodynamic and sustainability performance compared with conventional manually operated dryers. The findings suggest that the AFNSD, when optimized for product characteristics and loading configurations, offers a viable and energy-efficient solution for fruit drying in regions with high solar availability. Future work should focus on enhancing exergy efficiency through improved insulation and heat recovery, as well as testing with different agricultural products to broaden its application potential.

## Data Availability

All data are presented within the article.

## List of symbols

$$MC$$: Moisture content.
*W*: Sample weight
${\dot {m}_a}$: Mass flow rate
${\dot {E}_a}$: Energy flow rate
$${h_a}$$: Enthalpy
$${v_a}$$: Air velocity
$${z_a}$$: Height
*g*: Gravity acceleration
$$\dot {W}$$: Work done
$$\dot {Q}$$: Heat transfer
$${\dot {Q}_u}$$: Useful energy
$${\dot {Q}_{in}}$$: Input energy
$${\dot {Q}_{ls}}$$: Energy loss
${I_s}$: Solar radiation intensity
${A_{SAC}}$: Surface area of the solar collector
${C_{pa}}$: Specific heat of air
$${T_c}$$: Air temperature
$${\eta _{en}}$$: Energy efficiency
${m_w}$: Quantity of removed water from date sample
*L*: Latent heat of vaporization of water
$${t_d}$$: Drying time
$${\eta _{ex}}$$: Exergy efficiency
*u*: Internal energy
*s*: Entropy
$\alpha$: Absorptivity of glass
$$\mathop {Ex}\limits^{.}$$: Exergy
$\tau$: Transmissivity of glass
$${\mu _{ch}}$$: Chemical energy
$${T_0}$$: Atmospheric temperature

## Subscription

*i*: Inlet
*o*: Outlet
${\mathrm{SAC}}$: Solar air collector
Dryer: The solar dryer
DR: Drying room

## Abbreviations

AFNSD: Automated forced and natural solar dryer
CFD: Computational fluid dynamics
SC: Solar Collector
DCh: Drying chamber
ETSC: Evacuated tube solar collector
SRI: Solar radiation intensity
IP: Improvement potential
WER: Waste exergy ratio
SI: Sustainability Index
SD: Solar dryer
MC: Moisture content
PV: Photovoltaic
